# Time-division multiplexing for myoelectric closed-loop control using electrotactile feedback

**DOI:** 10.1186/1743-0003-11-138

**Published:** 2014-09-15

**Authors:** Strahinja Dosen, Marie-Caroline Schaeffer, Dario Farina

**Affiliations:** Department of Neurorehabilitation Engineering, University Medical Center Göttingen (UMG), Georg-August University, Göttingen, TX 37075 Germany; Ecole Centrale de Nantes, département Automatique et Robotique, Nantes, 44321 France

**Keywords:** Sensory feedback in prosthetics, Sensory substitution, Time-division multiplexing, Closed-loop control, Feedback window

## Abstract

**Background:**

Restoring sensory feedback in myoelectric prostheses is still an open challenge. Closing the loop might lead to a more effective utilization and better integration of these systems into the body scheme of the user. Electrotactile stimulation can be employed to transmit the feedback information to the user, but it represents a strong interference to the recording of the myoelectric signals that are used for control. Time-division multiplexing (TDM) can be applied to avoid this interference by performing the stimulation and recording in dedicated, non-overlapping time windows.

**Methods:**

A closed-loop compensatory tracking task with myocontrol and electrotactile stimulation was used to investigate how the duration of the feedback window (FW) influences the ability to perceive the feedback information and react with an appropriate control action. Nine subjects performed eight trials with continuous recording and contralateral feedback (CONT-CLT) and TDM with ispilateral stimulation and recording using the FW of 40 ms (TDM40), 100 ms (TDM100) and 300 ms (TDM300). The tracking quality was evaluated by comparing the reference and generated trajectories using cross-correlation coefficient (CCCOEF), time delay, root mean square tracking error, and the amount of overshoot.

**Results:**

The control performance in CONT-CLT was the best in all the outcome measures. The overall worst performance was obtained using TDM with the shortest FW (TDM40). There was no significant difference between TDM100 and TDM300, and the quality of tracking in these two conditions was high (CCCOEF ~ 0.95). The results demonstrated that FW duration is indeed an important parameter in TDM, which appears to have an optimal value. Among the tested cases, the FW duration of 100 ms seems to be the best trade-off between the quality of perception and a limited command update rate.

**Conclusions:**

This study represents the first systematic evaluation of a TDM-based approach for closing the loop using electrotactile feedback in myoelectric systems. The overall conclusion is that TDM is a feasible and attractive method for closed-loop myocontrol, since it is easy to implement (software-only solution), has limited impact on the performance when using proper FW duration, and might decrease habituation due to burst-like stimulation delivery.

**Electronic supplementary material:**

The online version of this article (doi:10.1186/1743-0003-11-138) contains supplementary material, which is available to authorized users.

## Background

An ideal human-machine interface between an amputee and a prosthesis should restore both feedforward and feedback communication pathways, so that the commands can be sent to the prosthesis and the system states fed back to the user[1]. It is known from the motor control studies that both of these channels are instrumental for achieving sensory-motor integration and thereby smooth and effective execution of human movements[2]. An intuitive feedforward interface for active prostheses can be realized using myoelectric control[3], in which the user intention is detected by recording the electrical activity of his/her muscles using electromyography (EMG). In a recent review[4], the users of myoelectric prostheses acknowledged that the provision of feedback is an important requirement that should be addressed in the future. Although closed-loop prosthetic devices have been described and tested in the past[5–7], these developments have been confined to the research laboratories, and there are still no commercially available myoelectric systems providing any kind of somatosensory feedback to the user.

Different methods can be used to transmit state variables (e.g., joint angles, contact forces) from a sensorized prosthesis to an amputee[8]. The general approach is known as sensory substitution since the missing sensory information is communicated by stimulating alternative sensory receptors instead of those lost due to an amputation[9]. The most common approach is to elicit tactile sensations over the skin of the residual limb. The tactile sense can be stimulated using direct mechanical stimulation (e.g., vibration motors[10], linear pushers[11]) or by delivering electrical current pulses[12–14] activating skin afferents (i.e., electrotactile stimulation). The latter approach has certain advantages: since there are no moving mechanical parts, the hardware is very compact, the response is fast (no inertial effects), the energy consumption is low, and the operation is silent. However, an important drawback for the practical implementation of the closed-loop control using electrotactile stimulation is the interference between the electrical stimulation and the EMG recording (myoelectric control). When electrical pulses are delivered while recording EMG, the electrical stimuli can saturate the amplifier and/or corrupt the recorded signals, producing artefacts that can be higher than the voluntary electrical muscle activity[15, 16]. The shape and amplitude of the artefacts depend on the setup parameters (e.g., current intensity, amplifier gain) and the relative positioning of the stimulation and recording electrodes. The interference can be decreased by placing the electrodes further apart and/or by lowering the stimulation intensity[17]. However, both of these methods could be difficult or even impossible to implement during practical applications. The space available for the electrode placement depends on the size of the residual limb, which in many cases can be rather short. The stimulation intensity has to be at the level that can be clearly perceived by the subject and it is often modulated throughout the entire dynamic range in order to best transmit the changing feedback information[18]. If the artefacts cannot be eliminated by using a specialized hardware (e.g., amplifier with a blanking input[19]) or by adjusting the setup of the closed-loop system, they can be suppressed to a certain extent using post-processing[15, 16].

Alternatively, the interference between the stimulation and recording could be avoided by operating the closed-loop system using a time-division multiplexing (TDM) mode. In TDM, the stimulation and recording are performed sequentially within dedicated, non-overlapping time windows. TDM has never been tested before for the control in closed-loop systems. The method has been mentioned as a possible solution[20] and one prototype system implementing TDM was presented[5], but there was no systematic evaluation of the approach. An important parameter in TDM is the duration of the feedback window (FW). Longer stimulation window might result in a better perception of the delivered stimulation but it also increases the delay in responding to the user command. The aim of this study was to evaluate how the FW duration influences the quality of perception of the feedback information and the ability of the subject to respond to this information with an appropriate control action. As experimental setup, we used a compensatory tracking task that has been routinely applied as the standard test bench for evaluating different aspects of closed-loop human control systems, including visual[21] as well as electrotactile feedback[22, 23].

## Methods

### Time-division multiplexing

**Figure 1:**
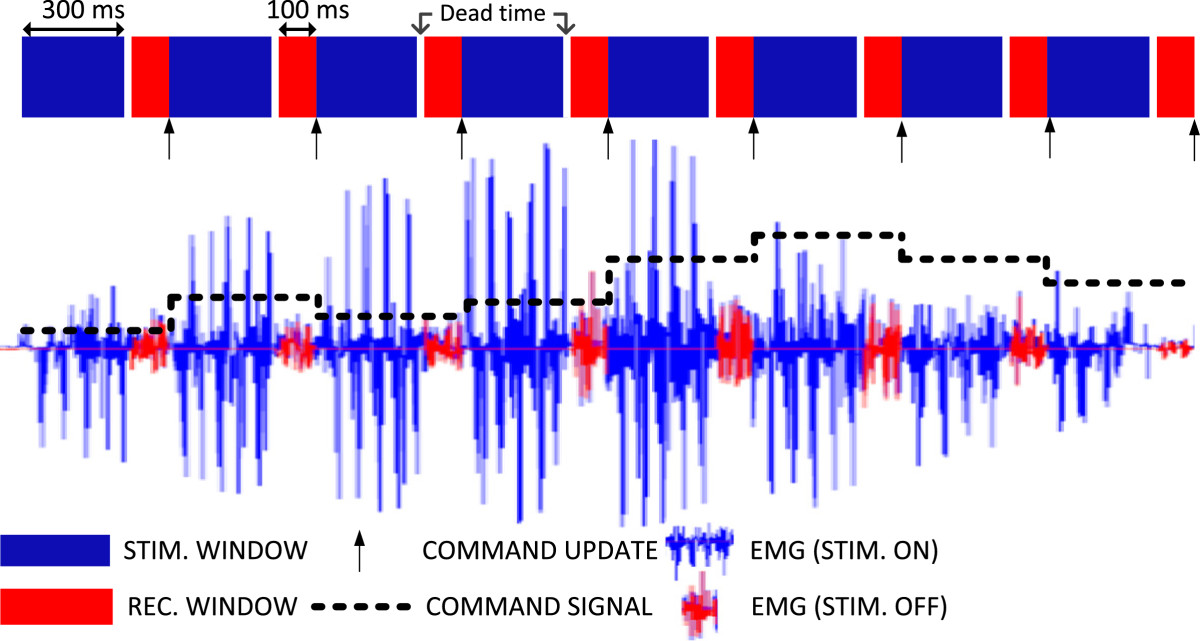
**Time-division multiplexing (TDM) for closed-loop myocontrol using electrotactile stimulation.** Recording (red) and stimulation (blue) windows were set to 100 and 300 ms in this particular example, respectively. The stimulation was delivered in bursts, producing large artefacts in the recorded EMG. Artefact-free EMG was collected during the recording intervals and processed to update the feedforward command (dashed black line) at the end of the window (i.e., root mean square of the recorded data). A brief 20-ms interval of a dead time (no recording and no stimulation) was inserted in-between the stimulation and recording (white zones) in order to allow the EMG amplifier an additional time to fully recover from the artefacts.

The TDM approach to closed-loop control multiplexes in time the recording and stimulation. During the recording interval, the stimulation is not delivered and artefact-free EMG is captured and processed to estimate the user command (feedforward). In the subsequent stimulation interval, the electrical pulses are delivered to convey the feedback information to the user. During stimulation, the recording is either paused by electrically disconnecting the amplifier inputs or it continues but the (corrupted) data are discarded (i.e., not used for prosthesis control). In the current study, the latter approach was used. Figure 1 shows that the electrical pulses delivered in bursts produced large artefacts in the recorded EMG during the stimulation windows. However, the EMG in-between the bursts was artefact-free (stimulation off), and thus the signal segments in these intervals (recording windows) were used to determine the control commands. More precisely, the feedforward command was updated at the end of the current recording interval and then it was held constant for the duration of the next stimulation and recording window. Therefore, with respect to continuous closed-loop control, in TDM the feedback was delivered intermittently while the command signal was discretized in time, comprising constant segments of equal durations. Duration of the stimulation (DSW) and recording windows (DRW) determined the three parameters characterizing the operation of TDM control loop: duration of the feedback burst (DSW), pause between the two feedback bursts (DRW), and command update rate (DSW + DRW). Effectively, the myocontrol in TDM implemented a sample (DRW) and hold (DSW + DRW) processing of the muscle activity.

### Experimental setup

**Figure 2:**
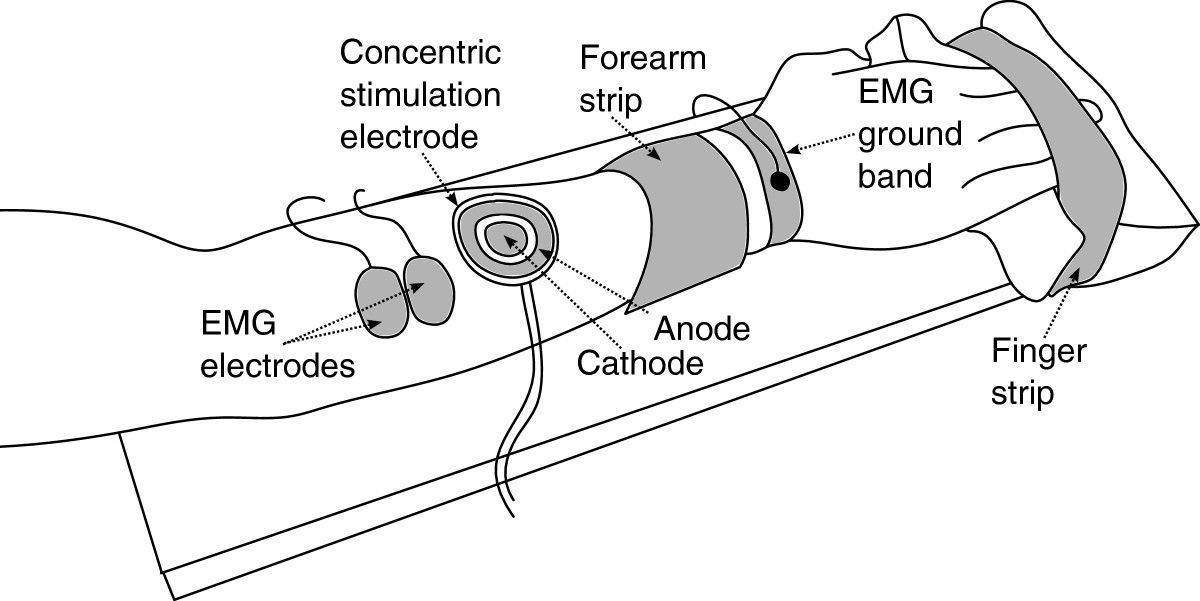
**Experimental setup.** The subject’s dominant hand was strapped to the wooden support. Two pairs of bipolar EMG electrodes were used to capture the activity of the wrist and finger extensor (shown) and flexor (not shown) muscles. Ground electrode was placed around the wrist. The subject controlled the closed-loop system by exerting nearly isometric contractions of the wrist and finger muscles. Two concentric electrodes were placed next to the recording ones to deliver the electrotactile feedback.

The experimental setup is shown in Figure 2. The subject’s dominant hand was positioned on the wooden support and strapped down firmly next to the wrist and around the fingers. A soft cushion was placed below the fingers. This setup allowed the subjects to produce nearly isometric contractions of the wrist and fingers without straining the finger joints. Two pairs of Ag/AgCl EMG electrodes (Neuroline 720, AMBU, US) were placed on the dorsal and volar side of the forearm, proximal to the elbow, to capture the activity of the wrist and finger flexor and extensor muscles. Before placing the electrodes, the skin was treated with a small amount of abrasive paste (everi, SpesMedica, IT). Two concentric stimulation electrodes (CoDe 2.0, OTBioelettronica, IT) were positioned just distally to the recording electrodes. This was done to emulate the likely positioning in real-life situations when the space available for electrode positioning may be very limited due to a short residual limb of an amputee. With this setup, the electrical stimulation significantly corrupted the recorded EMG signals. This was assessed in pilot tests, which also demonstrated that control was not feasible when the stimulation and recording were performed simultaneously, i.e., during compensatory tracking, the generated trajectory would irrecoverably diverge from the reference.

**Figure 3:**
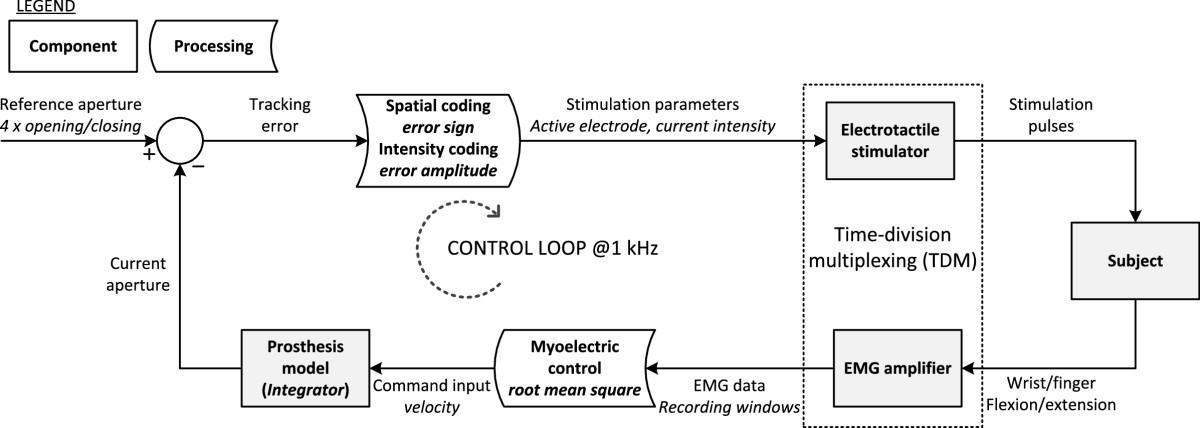
**The main components and processing blocks of the closed-loop control system for compensatory tracking using myocontrol and electrotactile feedback with time–division multiplexing (TDM).** The task for the subject was to steer the model of a prosthesis using isometric contractions so that the aperture follows a predefined trajectory. The feedback signal delivered using electrotactile stimulation was the current tracking error. The error information was mapped to stimulation through a combination of spatial (error sign → active electrode) and intensity (error amplitude → stimulation intensity) coding. The prosthesis was controlled using classic approach, i.e., the input command signal was proportional to the velocity of opening/closing.

The task for the subject was to control a virtual model of a prosthesis using myoelectric signals so that the prosthesis aperture followed a predefined reference trajectory (Figure 3). The current tracking error, the difference between the generated and reference trajectory, was fed back to the subject using electrotactile stimulation. The stimulation at the dorsal and volar electrode indicated positive and negative tracking error, respectively, while the intensity of the stimulation was proportional to the error amplitude. The goal was to compensate the error, a condition felt by the subject as a low stimulation intensity. When the subject felt dorsal stimulation, he/she activated flexors driving the prosthesis model in the proper direction to decrease the error, and analogously, to compensate for the volar stimulation, he/she contracted the extensor muscles.

The stimulation was delivered using a fully programmable multichannel unit (RehaStim, Hasomed, Gmbh) and the EMG signal was recorded using an analog amplifier (AnEMG12, OTBioelettronica, IT). The stimulator was connected to a desktop PC via USB port, and the channel activity and stimulation parameters were set online by sending simple textual commands at a rate of 50 Hz. The two bipolar channels of the EMG amplifier were connected to a DAQ card (NI PCI 6221, National Instruments, US) and sampled at 1 kHz. The control loop shown in Figure 3 was implemented in Matlab Simulink using Real Time Windows Target toolbox and therefore the system operated in real time at 1 kHz. An integrator (*5/s*, where *s* is the Laplace variable) was used as the model of the prosthesis. The input for the integrator was a normalized bipolar signal, setting the rate of change (velocity) of the integrator output. Maximum negative and positive velocity corresponded to -1 and 1 (a.u.), respectively, while zero value indicated no change (output stationary). Positive signal, increasing the output, was provided by the extensors and negative, decreasing the output, by the flexor muscles. The subject’s EMG therefore controlled proportionally the velocity of the prosthesis opening and closing, emulating the control that is commonly used in the real systems[24, 25]. To compute the input for the prosthesis model, the root mean square (RMS) of the EMG within the recording window was calculated and normalized to the maximum value registered during the calibration step (see Experimental protocol).

### Experimental protocol

The reference signal for the tracking was an 80-s long sequence comprising four step profiles (i.e., single step = baseline-plateau-baseline) of which two steps were larger with a normalized amplitude of 1 (a.u.) and two were smaller with an amplitude of 0.5 (a.u.). To minimize the effect of learning and predicting, the steps were randomized in order and sign. The rising and falling edges of the steps were slightly sloped producing a gradual increase in the stimulation intensity which was thus more comfortable for the subjects. The horizontal segments (i.e., step plateaus and signal baselines) of the reference were 8 s long. A single step profile was equivalent to the hand opening (from the baseline to the plateau) and closing (from the plateau back to the baseline); therefore, the reference trajectory comprised four sequences of opening and closing.

The experiment was organized in two sessions: introductory session (1 h) and test session (2 h 30 min), which were performed in two consecutive days. This structure was selected based on the pilot tests. To accomplish the task, the subjects had to familiarize with three different concepts: myocontrol, tracking task and electrotactile feedback. The last one in particular was especially challenging since using tactile feedback for conscious and fine online control is not common in daily life, i.e., visual feedback is preferred for such tasks. The pilot tests indicated that the subjects would be overwhelmed if both the introduction and main tests were to be performed within a single session.

At the beginning of each session, the myoelectric control was calibrated for each subject individually. The subjects were asked to perform four maximum flexions and extensions of the wrist and fingers as well as rest the hand following the visual cues. The collected calibration data were used to adjust the gains and dead zones for the EMG channels. For each muscle group (flexors/extensors), the calibration parameters were set so that the RMS of the EMG acquired during the recording window was mapped linearly to the interval between 0 and 1, as follows. When the subjects relaxed the muscles, the EMG RMS was below the dead zone threshold and thereby generated zero command. The RMS equal to 80% of the RMS registered during the maximal voluntary contractions (MVC) was mapped to one (maximum command). Therefore, to produce the maximum command, the subject had to generate a sustained contraction at the level of 80% of MVC during at least one recording window. Since the recording window was brief (100 ms) and allocated few times per second, the subjects were able to steer the signal comfortably throughout the full range. Finally, the flexor signal was multiplied by -1 and then added to the positive signal coming from the extensor, resulting in the bipolar input driving the prosthesis model. In each session and for each TDM condition (see below), the sensation (ST) and pain (PT) thresholds for electrotactile stimulation were determined for both electrodes using the method of limits[26]. The current amplitude and frequency were constant and set to 3 mA and 100 Hz, respectively, while the pulse width was modulated to regulate the intensity of stimulation. Previous experiments demonstrated that the stimulation with these parameters allowed good perception and modulation of the elicited tactile sensations[27]. Importantly, when determining the ST and PT for the TDM conditions, the stimulation was delivered as specified by the TDM parameters (see below). The tracking error in the interval [0, 1] was linearly mapped to the dynamic range which was defined as the interval between ST and 0.8 * PT.

The purpose of the introductory session was to familiarize the subjects to the electrotactile stimulation, myoelectric control, and compensatory tracking task. To this aim, the subject first tracked the aforementioned reference signal (four step profiles) using visual feedback (5 trials). In this condition, the tracking error was represented by a red sphere moving vertically on the screen (standard 22″ monitor). Initially, the sphere was positioned in the middle of the screen over an anchor point (zero error). When the trial started, the sphere moved away from the anchor proportionally to the tracking error. Positive and negative errors moved the sphere in the up and down direction, and in response the subject activated flexor and extensor muscles driving the system in the proper direction to cancel out the errors, respectively. Note that the visual setup was spatially consistent with the semantics of the electrotactile stimulation using dorsal (top ~ up) and volar (bottom ~ down) electrodes. Next, the subjects performed the same task but with the addition of electrotactile feedback (5 trials). Electrotactile stimulation was delivered continuously and to avoid the artefacts in the myoelectric signals, the concentric electrodes were placed onto the contralateral forearm. With the feedback in both modalities, the subjects could relate the tactile sensation to the visual feedback and thus learn to interpret the meaning of the electrotactile feedback. Finally, the subjects conducted 5 trials using continuous electrotactile feedback only. Between the trials the subjects were instructed on how to improve the performance (e.g., avoid abrupt, strong reactions typically leading to large oscillations around the reference, perceive the stimulation intensity and react proportionally etc.). The introductory session therefore gradually introduced the subjects to the experimental setup and tasks, serving also as the training for myoelectric visual and electrotactile tracking.

In the next session, the subjects performed electrotactile tracking tasks using continuous stimulation and recording (CONT-CLT) and TDM with three durations of the FW: 40 ms (TDM40), 100 ms (TDM100), and 300 ms (TDM300). A dead time of 20 ms (no stimulation and no recording) was inserted after each stimulation delivery in order to allow enough time for the stimulation artifacts to fade out completely from the recorded EMG. Eight trials were conducted in each condition. In all TDM conditions, the recording window was set to 100 ms. According to the aforementioned sample-and-hold paradigm (see "Time-division multiplexing"), the duration of the recording window was set to a small value assumed to give a reasonable estimate for the current level of muscle activity. The selected value was within the range used in myoelectric control[3], and it was also checked preliminary in the pilot tests. To implement CONT-CLT, two more electrodes were placed on the contralateral forearm to the same position as the electrodes used for TDM. The continuous stimulation and control served as the benchmark condition. Since the tracking tasks can be cognitively demanding, the performance might be influenced by mental fatigue (level of attention and focus). Therefore, regular breaks, 2–5 min between the trials and 5–10 min between the conditions, were provided throughout the sessions in order to allow the subjects to rest. The continuous stimulation and recording on the same forearm (i.e., CONT-IPSI) was not considered, since such setup would cause interference between the recording and stimulation. In the current study, only the configurations providing artifact-free control were of interest as practically viable solutions. Controlling a prosthesis in the presence of artifacts would be very difficult if not impossible, both in proportional mode (pilot tests with Otto Bock Michelangelo Hand[24]) as well as using pattern recognition[16].

Nine healthy able-bodied subjects (29 ± 7 yrs) participated in the experiment. The order of the conditions was randomized for each subject to account for the possible confounding effects, such as, the influence of learning, development of mental fatigue, and the changes overtime in the sensitivity to the electrotactile stimulation. Also, the stimulation thresholds were reevaluated in each condition before commencing the test trials, as explained before. The experiment was approved by the Ethics Committee of the University Medical Center Goettingen, and before the sessions, the subjects signed an informed consent form.

### Data analysis

To evaluate the control performance in each condition, several performance measures were used to assess the quality of tracking. First, the average time delay (TD) between the reference and generated signal was estimated by locating the maximum value of the cross-correlation function. All the rest outcome measures were calculated by using a time-shifted version of the generated signal, compensating for the estimated time delay. The average similarity in shapes between the reference and generated trajectory was determined by calculating the cross-correlation coefficient (CCCOEF), while the root mean square tracking error (RMSTE) was used to assess the average amplitude difference. Finally, the amount of overshoot defined as the maximum difference between the reference and generated signals over each of the horizontal segments (plateaus and baselines) was calculated.

Statistically significant differences in the mean performance between the conditions were tested using one-way repeated measures ANOVA and Tukey’s honestly significant difference test for the post-hoc pairwise comparisons. A separate ANOVA was performed for each outcome measure. In addition, the performance variability across subjects in each condition was also evaluated for the statistically significant differences. First, Levene multiple-sample dispersion test was applied to assess if there was a statistically significant difference within the set of all conditions. If the test indicated significance, the conditions were compared pairwise using F-test for equal variances evaluating a pair of conditions at a time with Bonferroni adjustment to account for multiple comparisons. The threshold for the statistical significance was adopted at p < 0.05.

## Results

**Figure 4:**
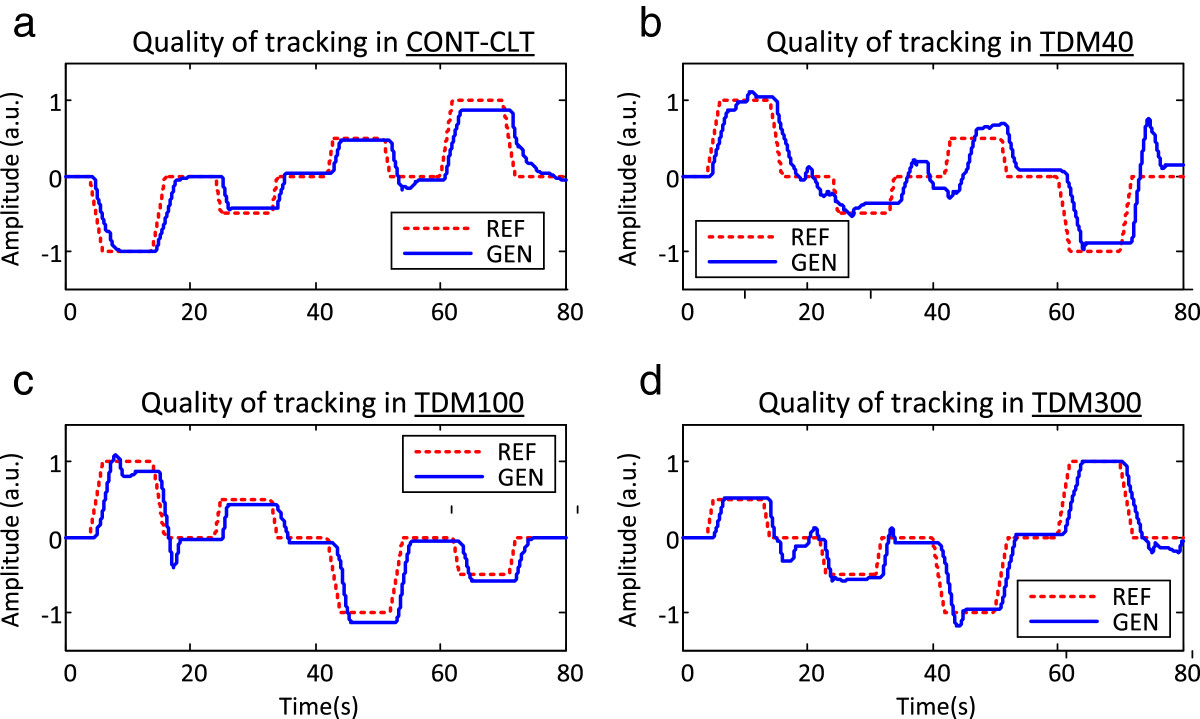
**The reference (red, dashed line) and generated (blue, solid line) trajectories for one subject in the four tested conditions.** The plots are for **a)** continuous feedback (CONT-CLT), and TDM using feedback window of **b)** 40 ms (TDM40), **c)** 100 ms (TDM100), and **d)** 300 ms (TDM300). The quality of tracking was the best in CONT-CLT, similar in TDM100 and TDM300, and much worse for the shortest feedback window in TDM40.

Figure 4 shows a representative tracking from one subject in each of the tested conditions. The best quality of tracking was achieved when the feedback was delivered with no interruptions (CONT-CLT). The generated trajectory closely followed the reference, i.e., all the major segments (baselines, slopes, and plateaus) were successfully reproduced (Figure 4[a]), although with a visible time delay. In addition, the generated slopes were less steep and the transitions between the slopes and baselines/plateaus more gradual. When the stimulation was delivered using TDM with the shortest window (TDM40), the performance worsened substantially (Figure 4[b]). In this condition, there were a general jerkiness and frequent overshoots in the generated trajectory, with an especially large deviation when coming back to the final baseline (blue signal peak at the end). The two middle steps, although small in amplitude, were tracked very poorly and were distorted substantially in the generated trajectory. For longer stimulation windows (TDM100 and TDM300), the quality of the tracking was similar to that achieved with the continuous stimulation. However, the performance was still worse than in CONT-CLT, and the tracking was characterized by overshoots as well as larger deviations from the reference.

**Figure 5:**
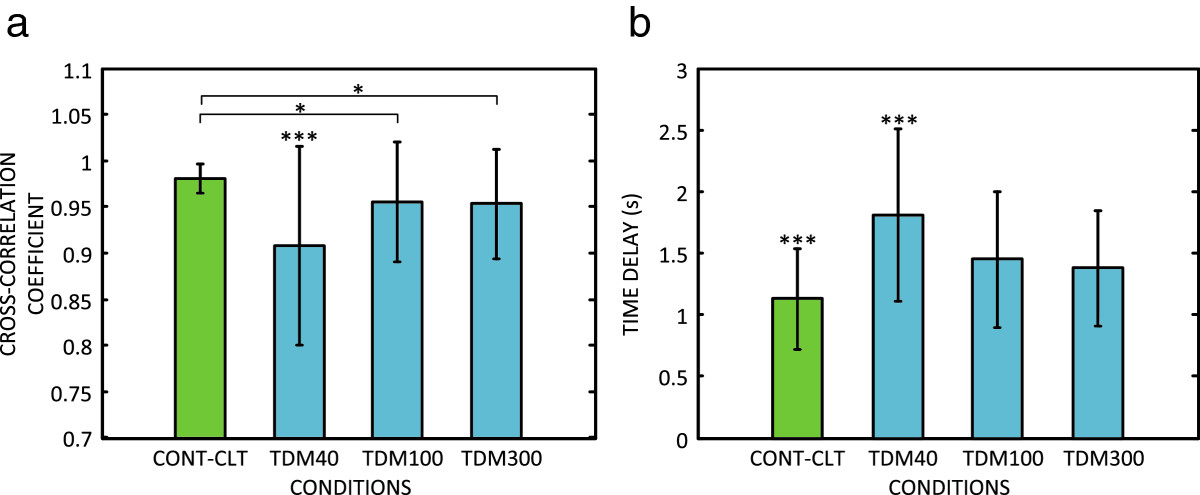
**Average results (mean ± standard deviation) across conditions: a) cross-correlation coefficient and b) time delay between the reference and generated trajectory.** Horizontal bar with asterisks indicates the statistically significant difference between the respective conditions. Asterisks only indicate that a group is significantly different from all the other conditions. Notation: CONT-CLT – continuous feedback, TDM40, TDM100, TDM300 – time–division multiplexing (TDM) with 40 ms, 100 ms, and 300 ms feedback window length. (*, p < 0.05; **, p < 0.01; ***, p < 0.001).

The average results across conditions are summarized in Figures 5 and6. Continuous feedback and control (CONT-CLT) resulted in the best performance in all the outcome measures. When the closed-loop control was implemented using TDM, CCCOEF decreased, while the time delay, RMSTE and overshoot increased. The worst performance overall was obtained when using the shortest stimulation window (TDM40). For all the outcome measures, except overshoot, TDM100 and TDM300 led to significantly better scores compared to TDM40. There was no statistically significant difference between TDM100 and TDM300. Although the tracking in TDM conditions was generally worse compared to CONT-CLT, the average CCCOEF was still above 0.9 (i.e., 0.91 in TDM40, and ~0.95 in TDM100 and TDM300). The average time delay increased by approximately 20% in TDM100 and TDM300 compared to CONT-CLT, and the increase in RMSTE was less than 0.1, i.e., lower than 10% of the reference trajectory maximum. For TDM40, these values were at least two (RMSTE) and three (time delay) times greater.

**Figure 6:**
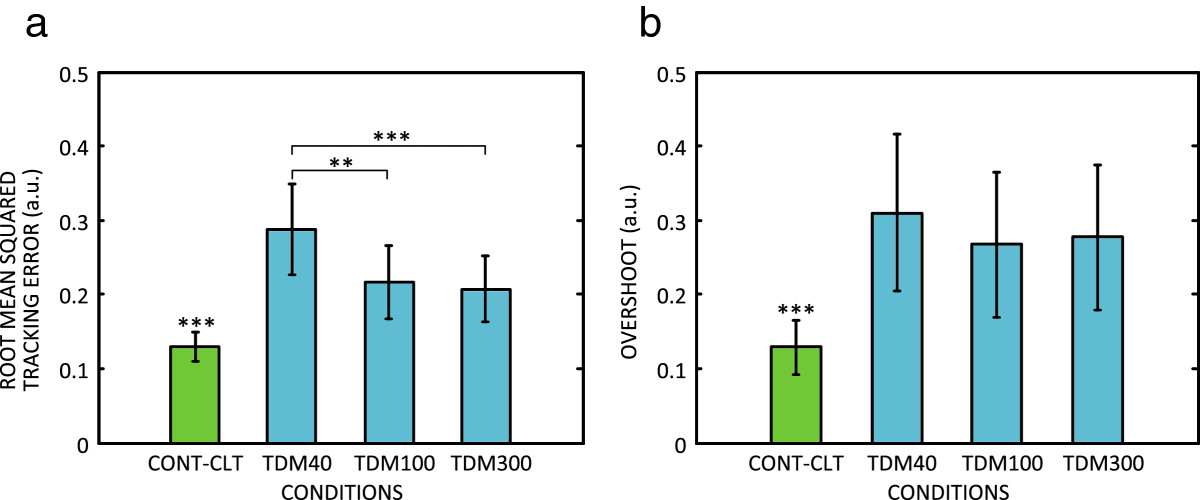
**Average results (mean ± standard error) across conditions: a) root mean square tracking error and b) overshoot between the reference and generated trajectory.** Horizontal bar with asterisks indicates the statistically significant difference between the respective conditions. Asterisks only indicate that a group is statistically significantly different from all the other conditions. Notation: CONT-CLT – continuous feedback, TDM40, TDM100, TDM300 – time–division multiplexing (TDM) with 40 ms, 100 ms, and 300 ms feedback window length. (*, p < 0.05; **, p < 0.01; ***, p < 0.001).

The performance during the continuous stimulation (CONT-CLT) was also most consistent across subjects. The variance in CONT-CLT was lower compared to the other conditions in all the outcome measures, and the differences were statistically significant. Within TDM, the results were significantly more variable in TDM40 compared to TDM100 and TDM300 for CCCOEF, and compared to TDM300 for the time delay and RMSTE.

## Discussion

We presented here the first systematic experimental evaluation of the TDM approach for closing the loop in human myoelectric control systems with electrotactile feedback. Closed-loop tracking was used as a standard test instrument, and the task for the subjects was to detect the tracking error (electrotactile stimulus), perceive its properties (e.g., amplitude), and respond with an appropriate compensatory control action by producing muscle contraction. The specific aim of the current study was to investigate how the duration of the feedback (stimulation) window (FW) affects the subjects’ ability to perceive the feedback and deliver the proper command, steering the simulated prosthesis back to the desired trajectory.

When the stimulation was delivered continuously to the contralateral forearm (CONT-CLT), the subjects were able to accomplish the task and achieve a good quality of tracking, with consistent results across subjects. This was a benchmark performance demonstrating what could be done when the recording for control and stimulation for feedback were simultaneous and uninterrupted. When using TDM to allow recording and stimulation on the same forearm, the performance generally decreased. The overall results indicated that the FW duration is indeed an important parameter, which seems to have an optimal value as explained below.

Considering the system responsiveness, the FW should be as short as possible, since during this period the user command signals (i.e., EMG) were not processed, i.e., the new value of the command input was computed only at the end of the subsequent recording interval (Figure 1). However, the experiments demonstrated that the subjects’ performance was the poorest in the case of the shortest FW (TDM40) (Figures 5 and6). With the FW of 40 ms, the control was updated and the feedback was delivered at a rate of about 6 times per second, which was the fastest refresh rate in the TDM conditions. Still, the individual stimulation bursts were rather short, comprising only 4 pulses, and it seems that this was not enough for the subjects to perceive the feedback information clearly. Therefore, they had to wait for several consecutive bursts before deciding on the control action, which produced long time delays (Figure 5[b]). When the stimulation bursts were prolonged to 10 pulses in TDM100, the perception and thereby the performance improved significantly. This was corroborated by the verbal feedback from all subjects who complained that they could not clearly feel the stimulation in TDM40 but not in the other conditions. However, increasing the duration of the feedback window further (TDM300) did not improve the results.

The current study therefore demonstrated that there could be an optimal FW duration for the implementation of the TDM. If the window is too short (TDM40), the performance decreases significantly, while prolonging the window above a certain limit does not lead to further improvements (TDM300). On the other hand, long windows introduce delays into the feedforward pathway, which has been shown to negatively affect the user experience and performance in prosthesis control[28]. According to this reasoning, the value of 100 ms could be regarded as the best trade-off between the feedforward time delay and the quality of perception among the considered TDM conditions. However, only three values were tested overall (due to time constraints), which were sufficient to determine a trend but not to precisely locate the actual optimal value. With the current setup, the accuracy of this estimate could be improved further by increasing the resolution and testing more values in the vicinity of this particular FW duration (100 ms).

The current result can be used as a promising starting point for the control of a real prosthesis using TDM approach. Still, it must be acknowledged that controlling a real system characterized with non-ideal dynamics (inertia, friction) and realistic interaction with the environment (e.g., impulsive contacts) represents a unique set of challenges. Therefore, the initial guess (100 ms) would have to be reevaluated, and an optimal FW duration could be in this context even task dependent (e.g., continuous force steering vs. abrupt contact detection). If the intensity coding is used, the implementation of TDM would be similar as in the current study. Each monopolar (e.g., aperture and force) and bipolar signal (e.g., velocity) would require one and two dedicated electrodes, respectively. The control task would be more difficult compared to the current study, since the subject would have to estimate the error signal using the feedback information (e.g., current grasping force) and desired goal (e.g., desired grasping force). Alternatively, the prosthesis variables can be represented using spatial coding over multiple electrodes. In this case, the current signal value is denoted by a currently active electrode within the array. This simplifies the application of TDM, since the user does not need to recognize the intensity (constant stimulation). The active electrode would flicker at a certain rate, but this is unlikely to affect the localization success rate substantially. An alternative approach to TDM, also of interest for practical application, would be to attempt the separation of the recording and stimulation in the frequency instead of the time domain. This could be implemented by delivering the stimulation at a frequency well above the bandwidth of interest for myocontrol.

To explain the current results, especially in TDM300, two distinct but still interrelated mechanisms have to be considered; the control performance could be affected by the quality of feedback perception as well as by the command update rate, where both of these were determined by the FW duration. In fact, how each of these factors contributed to the fact that the performance in TDM300 did not improve with respect to TDM100 cannot be deduced from the current experiment. It might be that increasing the FW duration from 100 to 300 ms did not improve the quality of perception significantly, as it did from 40 to 100 ms. Alternatively, the perception might have been better, but this could not be truly exploited due to the lowering of the command update rate. This points out to an inherent complexity of the TDM compared to the continuous control, since the former involves switching to both a discrete feedback and a time-discrete piecewise constant control. To fully understand the effects of each factor, they would have to be tested in isolation, e.g., continuous recording from one forearm and intermittent stimulation delivered contralaterally, and vice versa. However, this was outside the scope of the current study, and it might be anyway less relevant for the actual practical application (ispilateral stimulation and recording).

As explained before (see "Time-division multiplexing"), the duration of stimulation and recording windows both determine the operation of the TDM control loop. In the current study, only the former was changed across conditions, whereas the latter was set to a heuristically selected constant value (see "Experimental protocol"). Longer recording window might give a more reliable estimate of the current muscle activity, but it would also decrease the feedback and command update rates. Shorter window would speed up the control loop, but it might capture the momentary variations reflecting the noisiness of the EMG rather than the intended subject command. However, experimental validation of these predictions is needed.

The current setup considers only the control of prosthesis aperture but it could be extended to include grasping force. An additional pair of electrodes could be used to feedback the force information. Also, the prosthesis model would have to switch between the velocity control during free movement and proportional control during contact, since the grasping force is proportional to the command input[24, 25]. This is a very different system dynamics than the one tested in the current study and the performance of TDM is yet to be evaluated in this context.

The current study is the first systematic evaluation of the TDM approach for the closed-loop myocontrol. The aim was to test the aspect we deemed to be the most obvious and important for the control performance, i.e., the length of time during which the feedback was delivered at each update tick. However, there are other factors that could affect the control, but could not be investigated within a single experimental session. Some are specific to TDM and discussed above, and some are general confounding factors common to most studies using electrotactile stimulation (e.g., the choice of stimulation frequency, stimulation waveform shape, electrode size etc.). Importantly, although these factors could affect the exact values, they would not change the overall trends and insights determined here.

The results of the first experiments are rather encouraging. Although interrupting the afferent information stream and using a limited command update rate affected the performance, the impact was not substantial if the proper window duration was used (Figures 4 and5[a], TDM100 vs. CONT-CLT). On the other side, TDM is a simple, software-only solution to the problem of interference between the stimulation and recording, allowing the implementation of the closed-loop myocontrol with minimal effort. Furthermore, the suboptimal performance might be even more acceptable when taking into account that TDM would likely decrease habituation to electrotactile feedback since it delivers the stimulation intermittently, in bursts[29]. Therefore, the same method (TDM) could ideally be used to simultaneously address two challenges facing the closed-loop control, i.e., interference between recording and stimulation, and decrease in the feedback effectiveness due to the loss of sensitivity over time. Accordingly, the next steps in this research are to investigate if TDM indeed leads to a decrease in habituation with respect to the continuous feedback and to evaluate TDM as a method for online control of a real prosthesis. In both cases, the starting point for implementation will be the system presented here using the best FW duration (100 ms) as determined in the current study.

## Conclusions

This study demonstrated that TDM is a feasible method for closing the myocontrol loop using electrotactile stimulation to provide the feedback. The duration of the FW is an important parameter determining critically the quality of information perception and thereby the control performance. TDM is a simple method for avoiding the interference between the EMG recording and electrotactile stimulation. However, it limits the rate of command updates (feedforward) and also interrupts the afferent information stream (feedback). Nevertheless, if the FW duration is properly chosen, the impact of these limitations on the control performance is not substantial. In addition to the practicality, an advantage of TDM is that it can decrease sensory habituation due to burst-like (and not continuous) stimulation delivery.

## Authors’ original submitted files for images

Below are the links to the authors’ original submitted files for images. Authors’ original file for figure 1 Authors’ original file for figure 2 Authors’ original file for figure 3 Authors’ original file for figure 4 Authors’ original file for figure 5 Authors’ original file for figure 6

## Abbreviations

CCCOEF: Cross-correlation coefficient
CONT-CLT: Condition with the continuous stimulation (feedback)
FW: Feedback window
EMG: Electromyography
PT: Pain threshold
RMS: Root mean square
RMSTE: Root mean squared tracking error
ST: Sensation threshold
DSW: Duration of the stimulation window
DRW: Duration of the recording window
TDM: Time-division multiplexing
TDM40: Time division multiplexing with the FW duration of 40 ms
TDM100: Time division multiplexing with the FW duration of 100 ms
TDM300: Time division multiplexing with the FW duration of 300 ms.
