# Supplementary figures and images for: Time-division multiplexing for myoelectric closed-loop control using electrotactile feedback
Source: J Neuroeng Rehabil. 2014 Sep 15;11:138. doi: 10.1186/1743-0003-11-138 (PMC4182789; doi:10.1186/1743-0003-11-138)

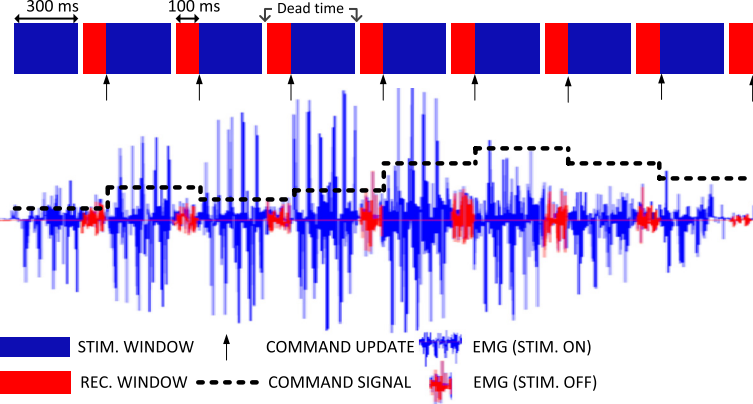

Supplement: Supplementary file 1 — Authors’ original file for figure 1 [file 12984_2014_659_MOESM1_ESM.pdf]

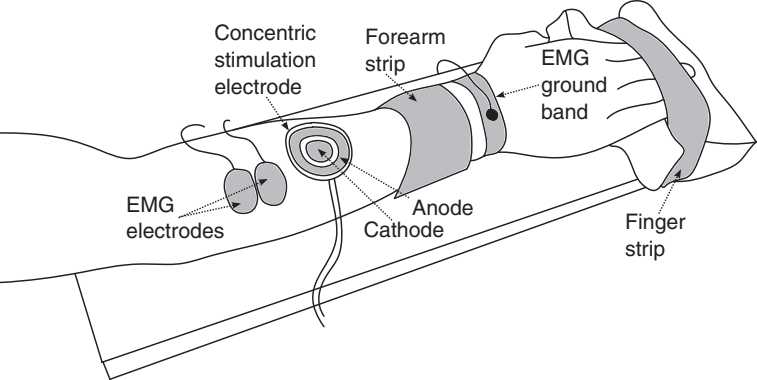

Supplement: Supplementary file 2 — Authors’ original file for figure 2 [file 12984_2014_659_MOESM2_ESM.pdf]

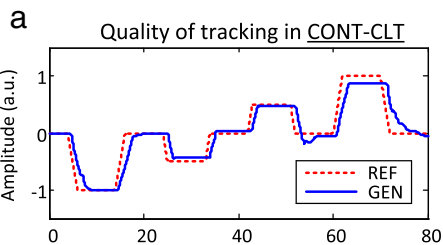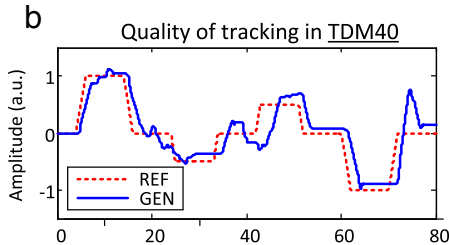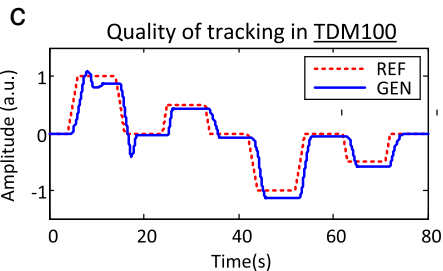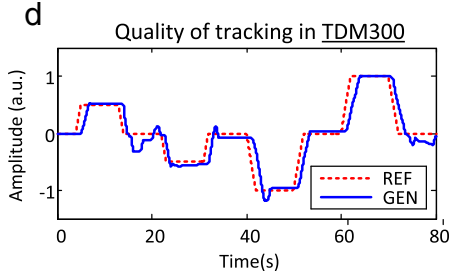

Supplement: Supplementary file 4 — Authors’ original file for figure 4 [file 12984_2014_659_MOESM4_ESM.pdf]

**a**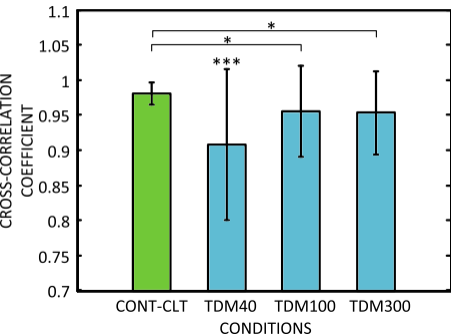**b**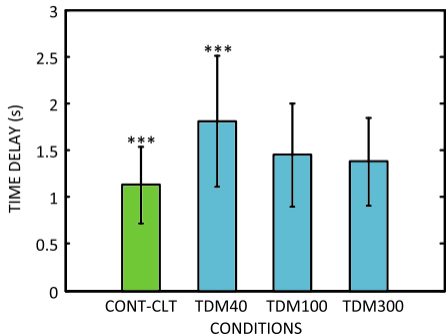

Supplement: Supplementary file 5 — Authors’ original file for figure 5 [file 12984_2014_659_MOESM5_ESM.pdf]

**a**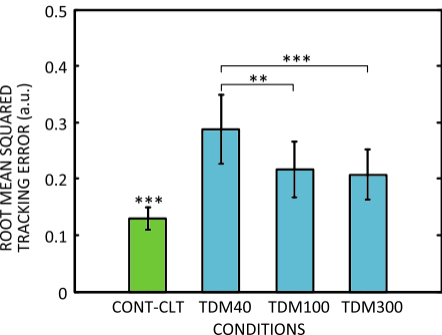**b**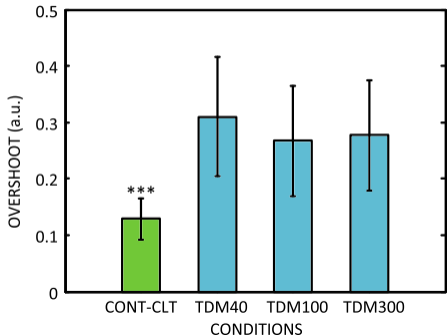

Supplement: Supplementary file 6 — Authors’ original file for figure 6 [file 12984_2014_659_MOESM6_ESM.pdf]
