# Supplementary material for: Time-division multiplexing for myoelectric closed-loop control using electrotactile feedback
Source: J Neuroeng Rehabil. 2014 Sep 15;11:138. doi: 10.1186/1743-0003-11-138 (PMC4182789; doi:10.1186/1743-0003-11-138)
Supplement: Supplementary file 3 — Authors’ original file for figure 3 [file 12984_2014_659_MOESM3_ESM.pdf]

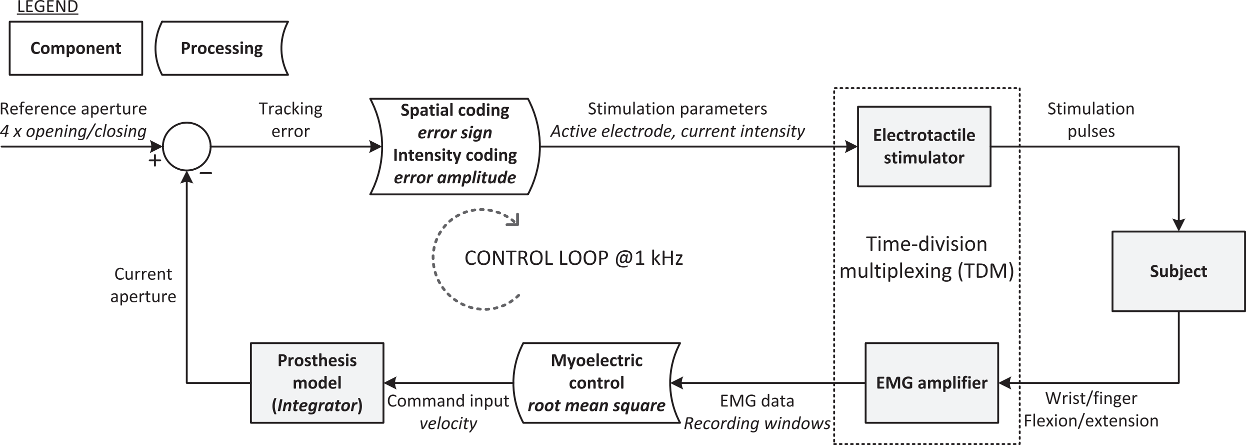
LEGEND
Component
Processing
Reference aperture
4 x opening/closing
+
−
Tracking
error
Spatial coding
error sign
Intensity coding
error amplitude
Stimulation parameters
Active electrode, current intensity
Electrotactile
stimulator
Stimulation
pulses
Subject
CONTROL LOOP @1 kHz
Time-division
multiplexing (TDM)
Current
aperture
Prosthesis
model
(Integrator)
Command input
velocity
Myoelectric
control
root mean square
EMG data
Recording windows
EMG amplifier
Wrist/finger
Flexion/extension
